# Correlation analysis of preoperative serum alpha-fetoprotein (AFP) level and prognosis of hepatocellular carcinoma (HCC) after hepatectomy

**DOI:** 10.1186/1477-7819-11-212

**Published:** 2013-08-27

**Authors:** Wen-jun Ma, Hai-yong Wang, Li-song Teng

**Affiliations:** 1 Cancer Center, The First Affiliated Hospital of Zhejiang University School of Medicine, Hangzhou 310003, P.R. China; 2 Hangzhou Xixi Hospital, Hangzhou 310023, P.R. China

**Keywords:** Alpha-fetoprotein, Hepatocellular carcinoma, Differentiation degree, Hepatectomy, Prognosis

## Abstract

**Background:**

To investigate the prediction value of preoperative serum alpha-fetoprotein (AFP) level for the prognosis of hepatocellular carcinoma (HCC), by comparing pathological characteristics, recurrence rate and survival rate after hepatectomy.

**Methods:**

108 cases of HCC patients who received liver resection in our hospital from 2005 to 2011 were enrolled in this study. According to preoperative serum AFP level, the patients were divided into AFP ≤ 20 ng/mL group, AFP 20 to 400 ng/mL group and AFP > 400 ng/mL group, and the clinicopathological and cytopathological features were compared. All the patients were followed up for 24 months, the postoperative recurrence rates and survival rates were compared and analyzed, and the risk factors for HCC postoperative survival rate were studied by multifactor regression analysis.

**Results:**

Of the 108 cases of HCC patients, there were 42 cases in AFP ≤20 ng/mL group, 28 cases in AFP 20–400 ng/mL group and 39 cases in AFP > 400 ng/mL group. It was shown that cell differentiation degrees (*χ*^2^ = 20.198, *P* = 0.000) and microvascular invasion rates (*χ*^2^ = 20.358, *P* = 0.000) were significantly different among the three groups. The AFP ≤ 20 ng/mL group showed higher cell differentiation degrees and significantly lower microvascular invasion rates compared to the other groups (*P* < 0.05). The follow-up data showed that postoperative 2-year recurrence rate (*χ*^2^ = 6.164, *P* = 0.046), 18-month survival rate (*χ*^2^ = 7.647, *P* = 0.022) and 24-month survival rate (*χ*^2^ = 6.725, *P* = 0.035) of the three groups were significantly different, and we found that the AFP ≤ 20 ng/mL group had lower postoperative 2-year recurrence rate, and higher 18-month survival rate and 24-month survival rate than the other two groups (*P* <0.05). Multiple logistic regression analysis indicated that tumor diameter (≥ 5 cm) and preoperative serum AFP level (> 400 ng/mL) were closely correlated with HCC postoperative survival rate (*P* <0.05).

**Conclusions:**

It is shown that preoperative serum AFP level has considerable predictive value for the malignant feature and prognosis of HCC. It is suggested that HCC patients with no contraindication of operation and serum AFP ≤ 20 ng/mL can benefit most from primary treatment of hepatectomy. While HCC patients with serum AFP higher than 20 ng/mL need comprehensive therapy besides surgical resection and close follow up.

## Background

Primary liver cancer is one of the common malignant tumors in China and is the second leading malignant tumor with high mortality rates [1]. There are three main types, namely, hepatocellular carcinoma (HCC), bile duct carcinoma and mixed-type HCC, and more than 90% are HCC. Alpha-fetoprotein (AFP) is a glycoprotein, mainly from the yolk sac and embryo liver; serum AFP levels are very low in adults. Since the 1970s, AFP has been used as a tumor marker for the diagnosis of HCC. Serum AFP levels in nearly 75% of cases of HCC are higher than 10 ug/L [2]. Serum AFP lever is still regarded as the most important serum marker for HCC diagnosis today, though it can be high in some non-cancerous liver disease and can be at a low level in some HCC patients [3,4]. In patients with cirrhosis or chronic hepatitis B or hepatitis C infections, AFP is the most important serum marker to predict liver cancer occurrence [5,6]. The serum AFP level not only has diagnostic value but also has predictive value for the prognosis of HCC. As a relatively cheap and mature method, serum AFP has been regarded as an important indicator of postoperative HCC recurrence and metastasis [7]. In addition, a high serum AFP level has been associated with larger tumor size, bilobar involvement, massive or diffuse-type tumors, and portal vein tumor thrombus [8]. Nevertheless, no consistent correlation has been established between serum AFP level and tumor stage, degree of tumor differentiation, or extrahepatic metastasis [9]. In this study, we analyzed the correlation of preoperative serum AFP levels with HCC malignant features and survival after hepatectomy, through 24 months of follow up of 108 patients with HCC, who underwent hepatectomy at a single center.

## Methods

### Ethical approval

This study was approved by the institutional review board (IRB) of the first affiliated hospital of Zhejiang University.

### Patient selection

Patients who underwent hepatectomy for primary liver cancer (n = 108) were enrolled in this study. Written informed consent for each patient was given before participating in the study. All patients were diagnosed as primary HCC on both imaging and histopathological study. All the histopathological diagnoses were confirmed to be HCC with distinctive microscopic features and immunohistochemistry staining results. Patients with serious heart, lung, kidney, or blood diseases, autoimmune liver disease, or the presence of other malignant tumors, were excluded. All the patients underwent surgical resection as their initial treatment. Radical resection was performed in patients with a regional tumor, and patients with a portal vein tumor thrombus received tumor resection plus portal vein thrombectomy or postoperative portal vein perfusion chemotherapy.

### Preoperative and postoperative tumor assessment

Preoperative tumor staging was performed by dynamic computed tomography (CT), assessing tumor size, tumor number, portal vein involvement and regional tumor invasion. Postoperative tumor assessment was made by pathological study of the degree of tumor differentiation, vascular involvement, nerve involvement, lymphatic involvement and non-cancerous liver tissue fibrosis. Adjacent non-cancerous liver tissue was examined by reticular fiber staining, according to the Ishak scoring system (1995) for scoring the degree of fibrosis. Histopathological immunohistochemistry staining of hepatocytes and CK7 were studied by using the EnVision (Zhongshan biotechnology, Beijing) two-step method.

### Measurement of serum AFP

Serum AFP concentrations were determined within 1 month before surgery using a commercially available chemiluminescence immunoassay kit. According to preoperative serum AFP levels, the patients were divided into three groups: (1) AFP negative, AFP ≤ 20 ng/mL (n = 41); (2) AFP low, AFP 20 ng/mL to 400 ng/mL (n = 28); (3) AFP high, AFP > 400 ng/mL (n = 39).

### Follow up

All the enrolled patients were followed up for 2 years. The postoperative recurrence rate was assessed at 1 and 2 years, and survival was assessed at 6, 12, 18, and 24 months.

### Statistical analysis

All the data were statistically analyzed using the SPSS 15.0 software. Quantitative data were presented as mean ± SD (x ± s). The ages and diameters of tumors in the three groups of patients were compared by analysis of variance; the rate and constituent ratio were compared by the chi-square test; comparisons between two of the three groups with performed by the Student-Newman-Keuls method. Multiple logistic regression analyses were performed to ascertain the independent risk factors for HCC postoperative prognosis. For study time points (t_K_) of 6, 12, 18, and 24 months after surgery:

Survival rate = Number of survival cases at t_K_ time point/Total number in study.

## Results

### Patients’ characteristics

The 108 HCC patients were divided into three groups according to their preoperative serum AFP levels. Age, gender, original liver disease and family history of liver disease did not differ between the three groups (*P* > 0.05), as shown in the Table 1.

**Table 1:** Clinicopathologic variables in 108 patients with hepatocellular carcinoma

**Variable**	**AFP negative**	**AFP low**	**AFP high**
Age at diagnosis, years, mean ± SD	52.5 ± 8.6	52.1 ± 7.8	52.7 ± 6.9
Gender, male/female	34/7	20/8	34/5
HBsAg-positive	37	27	38
Family history of liver cancer	6	3	8
Family history of HBV infection	11	7	16
No family history of liver disease	24	18	15
Tumor number =1	35	21	28
Tumor number ≥2	6	7	11
Tumor diameter, cm, mean ± SD	5.9 ± 6.9	5.2 ± 4.5	5.0 ± 3.9
Portal vein tumor thrombus			
No	37	23	33
Yes	4	5	6
Adjacent tissue invasion			
No	39	24	34
Yes	2	4	5
Lymph node invasion			
No	39	24	37
Yes	2	4	2
Cell differentiation^*^			
Well-differentiated	14	1	1
Moderate	24	25	33
Poor	3	2	5
Microscopic invasion^*^			
No	25	7	8
Yes	16	21	31
Nerve invasion			
No	39	25	39
Yes	2	3	0
Lymph node metastasis			
No	39	27	37
Yes	2	1	2
Fibrosis			
0 to 4 points	2	3	2
5 to 6 points	39	25	37
Recurrence rate, n (%)			
1-year	7 (17.1)	6 (21.4)	10 (25.6)
2-year^*^	8 (19.5)	7 (25.0)	18 (46.2)
Survival rate, n (%)			
6-month	38 (92.7)	25 (89.3)	33 (84.6)
12-month	35 (85.4)	24 (85.7)	28 (71.8)
18-month^*^	35 (85.4)	22 (78.6)	23 (59.0)
24-month^*^	33 (80.5)	20 (71.4)	21 (53.8)

Results are presented as number of patients unless stated otherwise. *P <0.05. AFP, alpha-fetoprotein.

### Preoperative clinicopathologic variables

The clinicopathologic variables in the three groups of patients, including tumor volume, total tumor diameter, portal vein tumor thrombus, adjacent tissue invasion, and lymph node involvement, were analyzed by statistical software. None of the variables above were statistically different (*P* > 0.05), as shown in the Table 1.

### Pathological features

The difference in the degree of tumor differentiation and vascular involvement were evaluated by the chi-square test. Both HCC tissue differentiation (χ^2^= 20.198, *P* = 0.000) and vascular involvement were statistically different (χ^2^ = 20.358, *P* = 0.000) among the three groups of patients. Through paired comparison, the differences between the AFP-negative and AFP-low group, and between the AFP-negative and the AFP-high group were both statistically significant (*P* < 0.05), whereas the difference between the AFP-low and AFP-high groups was not (*P* > 0.05), as shown in Table 2. Differences in nerve involvement, lymphatic involvement, and para-cancerous liver fibrosis, were not statistically significant (*P* > 0.05), as shown in Table 1.

**Table 2:** Paired comparisons between three groups of patients with hepatocellular carcinoma

**Comparison content**	**Test statistic**	**AFP-negative versus AFP-low group**	**AFP-negative versus AFT-high group**	**AFP-low versus AFP-high group**
Cell differentiation	*q*	3.95	5.56	1.47
	*P*	<0.05	<0.05	>0.05
Microscopic invasion	*q*	4.76	5.89	0.61
	*P*	<0.05	<0.05	>0.05
18-month survival rate	*q*	1.03	3.74	2.36
	*P*	>0.05	<0.05	>0.05
2-year survival rate	*q*	0.92	3.57	2.31
	*P*	>0.05	<0.05	>0.05
2-year recurrence rate	*q*	0.45	3.32	2.47
	*P*	>0.05	<0.05	>0.05

AFP, alpha-fetoprotein.

### Follow up

All patients received consecutive follow up for 2 years. One-year recurrence rates between the three groups of patients showed no statistical difference (χ^2^ = 0.448, *P* = 0.799), however, 2-year recurrence rates between the three groups, showed statistically differences (χ^2^ = 6.164, *P* = 0.046), as shown in Table 1. Two-year recurrence rates were compared in the three groups; between the AFP-negative and AFP-low groups, and the AFP-high and AFP-low groups, the differences were not statistically significant (*P* > 0.05); between the AFP-negative and the AFP-high group the difference was statistically significant (*P* < 0.05), as shown in Table 2. In all the 108 patients who underwent hepatectomy, 33 patients had recurrence within 2 years. Among them, six patients had a second surgical excision, seven had microwave ablation therapy, eighteen had trans-hepatic arterial chemotherapy and embolization, and two patients had no further treatment.

**Figure 1:**
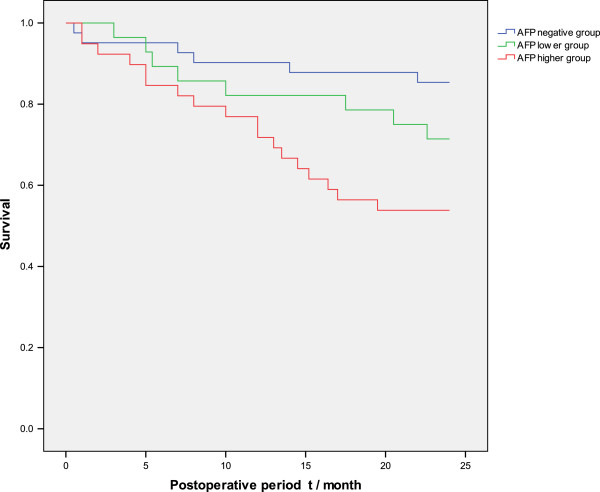
**Postoperative survival curve for three groups of patients with hepatocellular carcinoma.** AFP, alpha-fetoprotein.

The Kaplan-Meier method was employed to analyze the correlation between preoperative serum AFP levels and the prognosis of HCC. The survival rates in the three groups of patients at 6, 12, 18, and 24 months are shown in Table 1, and the survival curves in Figure 1. Our results show that higher preoperative AFP levels correlate with poorer prognosis. Among them, the survival rates at 18 months (χ^2^ = 7.647, *P* = 0.022) and 24 months (χ^2^ = 6.725, *P* = 0.035) were statistically different between the three groups. On paired comparisons, the differences were not statistically significant (*P* > 0.05) in the AFP-negative compared to the AFP-low group, or in the AFP-low compared to the AFP-high group, whereas the difference was statistically significant (*P* < 0.05) in the AFP-negative compared to the AFP-high group, as shown in the Table 2.

### Multivariate analysis

We performed univariate analysis of risk factors for HCC postoperative recurrence and survival, including age, gender, HBsAg-positive status, family history of liver cancer, degree of fibrosis, tumor volume, tumor diameter, and AFP level. Only tumor diameter and AFP level were statistically significant (*P* < 0.05), as shown in Table 3. Moreover, on multiple logistic regression analysis, tumor diameter and preoperative serum AFP levels were closely correlated with HCC postoperative survival rates (*P* < 0.05), as shown in Table 4.

**Table 3:** Univariate analysis for hepatocellular carcinoma postoperative prognosis

	**Recurrence (*P*)**	**Overall survival (*P*)**
Age at diagnosis, years	0.971	0.198
Gender, male versus female	0.056	0.701
HBsAg, negative versus positive	0.088	0.102
Family history of liver cancer, no versus yes	0.442	0.322
Tumor number, 1 versus ≥ 2	0.030	0.150
Tumor diameter, <5 versus ≥ 5 cm^*^	0.029	0.004
Fibrosis, 0 to 4 points versus 5 to 6 points	0.717	0.837
AFP, ≤ 400 versus > 400 ng/mL^*^	0.024	0.009

AFP, alpha-fetoprotein. ^*^P <0.05.

**Table 4:** Multiple regression analysis for hepatocellular carcinoma postoperative prognosis

	**Recurrence**		**Overall survival**	
	***P***	**HR (95% CI)**	***P***	**HR (95% CI)**
Tumor diameter, <5 versus ≥ 5 cm	0.039	3.787(1. 172 ~ 13.343)	0.021	4.423(1.353 ~ 17.356)
AFP, ≤ 400 versus >400 ng/mL	0.044	2.153(1.121 ~ 10.354)	0.035	3.875(1.207 ~ 15.573)

## Discussion

Primary liver cancer is a common malignant tumor. In many countries, including China, the incidence, recurrence and mortality rates of liver cancer remain high. Clinical studies have shown that there is a close relationship between the level of serum AFP and HCC incidence, recurrence and metastasis, and serum AFP level has been used as the main index of prediction for HCC prognosis after hepatectomy [5,10,11].

From pathological studies, there is literature showing that in normal liver tissue and serum AFP-negative HCC, AFP and AFP receptors are not expressed; AFP receptors are expressed only in the AFP-positive HCC tissues [12]. Other scholars conducted a comparative study of the ultrastructural and immunohistochemical features of the AFP-negative and -positive HCC tissues [13], and found the TN (Thomsen-Friedenreich-related antigen) protein expression level and positive rate in an AFP-negative group was significantly higher than in an AFP-positive group. The expression of AFP in the AFP-negative group was significantly lower than in the AFP-positive group. Under the electron microscope, AFP-negative HCC cells show simple organelles and rich free ribosomes; in AFP-positive ones, rich organelles, particularly the rough endoplasmic reticulum, and mitochondria and Golgi complex can be clearly observed in the cytoplasm. Whether these features are associated with AFP expression in HCC needs further study.

Through the retrospective study on the 108 patients with HCC, we found that the degree of differentiation in the AFP-negative group was higher than that in the AFP-low and the AFP-high groups, and the vascular infiltration rate in the AFP-negative group was lower than that in the other two groups, though the differences were not statistically significant between the AFP-low group and AFP-high group. Tumor size, number of portal vein tumor thrombi, adjacent organ invasion and lymph node involvement were not significantly different among the three groups. However, the above pathological characteristics were less severe in terms of malignant features in the AFP-negative than in the AFP-positive group, which might explain its better prognosis. On follow up, there were no significant differences in recurrence or survival rates between the three groups during the first 12 months. On 24-month follow up, patients in the AFP-negative group had a lower recurrence rate and higher survival rate when compared with the AFP-high group; however, the differences between the AFP-low and the AFP-high group were not significant. We also observed remarkable pathologically different features in the AFP-negative compared to the AFP-low group; however, the recurrence rate and survival rate were not significantly different. Our results showed that patients who had higher preoperative AFP levels were more likely to experience recurrence on short-term follow up. We need longer a follow-up period to confirm the long-term prognosis.

## Conclusions

In summary, based on the results of the current study, we revealed that the extent of malignancy and long-term recurrence rate of AFP-negative HCC is lower, survival rate is higher, and prognosis is better. Therefore, preoperative serum AFP level has considerable predictive value for the malignant features and prognosis for patients with HCC. Meanwhile, multiple logistic regression analysis indicated that preoperative serum AFP level (> 400 ng/mL) was an independent prognostic factor for HCC postoperative survival rates. Results from other studies also showed the AFP level was an important prediction factor for the recurrence and prognosis of HCC after resection, transarterial chemoembolization (TACE) or radiofrequency ablation (RFA) treatment [6,14-16], and it could be used to evaluate the prognosis of HCC as an independent influential factor [17,18].

For years, radical hepatectomy has been considered the standard treatment for resectable primary carcinoma [19]. From the results of this study, hepatectomy was shown to be less effective in HCC patients with higher preoperative AFP levels (> 400 ng/mL) compared to the AFP-negative patients, and similar results were reported in other studies [12]. Therefore, comprehensive therapy, needs to be considered as well as surgery, in patients with HCC and higher AFP levels (> 400 ng/ml); a combination with postoperative TACE or portal vein chemotherapy may lead to a better prognosis [12]. On the other hand, for the HCC patients with AFP levels between 20 and 400 ng/mL, the extent of malignancy is higher than that in AFP-negative patients, and long-term prognosis after resection need to be further studied; our group will continue to follow up these patients. Close follow up is needed of patients with preoperative AFP levels higher than 20 ng/mL.

In addition, in this study we found the degree of differentiation of AFP-negative HCC is relatively higher, and microscopic vascular involvement is less common. In the AFP-negative HCC the rate of tumor growth would probably be expected to be relatively slow and tumor staging might be lower than in AFP-positive HCC. However, at the time of clinical diagnosis of liver cancer, the staging of AFP-negative HCC was tantamount to that in AFP-positive HCC patients, which may be related to the lack of extensive use of a highly sensitive screening index. The use of a sensitive serum biomarker to detect the early recurrence and metastasis of AFP-negative HCC is rare before signs are apparent on imaging studies. It was reported that to some extent, high-sensitivity AFP-L3, AFP-IgM complex, calcium, GP73, DCP bursin, VEGA, GPC-3, and other markers can improve the sensitivity for detection of tumor recurrence in AFP-negative HCC [20-23], and have predictive value for the HCC therapeutic effect [24]. However, they have not been applied widespread because of the limitations in the technique and the cost of detection. As a result, novel serum markers that can predict recurrence in AFP-negative HCC need further investigation.

## Abbreviations

AFP: Alpha-fetoprotein; CT: Computed tomography; HCC: Hepatocellular carcinoma; RFA: Radiofrequency ablation; TACE: Transarterial chemoembolization.

## Competing interests

The authors declare that they have no competing interests.

## Authors’ contributions

MW carried out the follow up and performed the statistical analysis. WH drafted the manuscript. TL conceived of the study, and participated in its design and coordination. All authors read and approved the final manuscript.

## References

[B1] Gao JD Shao YF Xu Y Ming LH Wu ZY Liu GT Wang XH Gao WH Sun YT Feng XL Liang LM Zhang YH Sun ZT Tight association of hepatocellular carcinoma with HBV infection in North China Hepatobiliary Pancreat Dis Int 2005 11 46 49 15730918

[B2] Johnson PJ Role of alpha-fetoprotein in the diagnosis and management of hepatocellular carcinoma J Gastroenterol Hepatol 1999 11 32 36 10.1046/j.1440-1746.1999.01814.x 10382636

[B3] Tateishi R Yoshida H Matsuyama Y Mine N Kondo Y Omata M Diagnostic accuracy of tumor markers for hepatocellular carcinoma: a systematic review Hepatol Int 2008 11 17 30 10.1007/s12072-007-9038-x 19669276 PMC2716871

[B4] Snowberger N Chinnakotla S Lepe RM Peattie J Goldstein R Klintmalm GB Davis GL Alpha fetoprotein, ultrasound, computerized tomography and magnetic resonance imaging for detection of hepatocellular carcinoma in patients with advanced cirrhosis Aliment Pharmacol Ther 2007 11 1187 1194 10.1111/j.1365-2036.2007.03498.x 17944733

[B5] Baig JA Alam JM Mahmood SR Baig M Shaheen R Sultana I Waheed A Hepatocellular carcinoma (HCC) and diagnostic significance of A-fetoprotein (AFP) J Ayub Med Coll Abbottabad 2009 11 72 75 20364746

[B6] Lee HY Jung JH Kang YS Kim YS Moon HS Park KO Lee YS Kim SM Seo SW Lee SW Kim SH Lee BS Kim NJ Clinical significance of transiently elevated serum AFP level in developing hepatocellular carcinoma in HBsAg positive-liver cirrhosis [Article in Korean] Korean J Gastroenterol 2004 11 252 259 15100489

[B7] Chang SK Hlaing WW Yu RQ Lee TW Ganpathi IS Madhavan KK Value of alpha-foetoprotein for screening of recurrence in hepatocellular carcinoma post resection Singapore Med J 2012 11 32 35 22252180

[B8] Tangkijvanich P Anukulkarnkusol N Suwangool P Lertmaharit S Hanvivatvong O Kullavanijaya P Poovorawan Y Clinical characteristics and prognosis of hepatocellular carcinoma: analysis based on serum alpha-fetoprotein levels J Clin Gastroenterol 2000 11 302 308 10.1097/00004836-200012000-00007 11129271

[B9] Qin LX Tang ZY The prognostic significance of clinical and pathological features in hepatocellular carcinoma World J Gastroenterol 2002 11 193 199 11925590 10.3748/wjg.v8.i2.193 PMC4658349

[B10] Tong MJ Hsien C Song JJ Kao JH Sun HE Hsu L Han SH Durazo FA Saab S Blatt LM Factors associated with progression to hepatocellular carcinoma and to death from liver complications in patients with HBsAg-positive cirrhosis Dig Dis Sci 2009 11 1337 1346 10.1007/s10620-009-0747-y 19242792

[B11] Chan SL Mo FK Johnson PJ Hui EP Ma BB Ho WM Lam KC Chan AT Mok TS Yeo W New utility of an old marker: serial alpha-fetoprotein measurement in predicting radiologic response and survival of patients with hepatocellular carcinoma undergoing systemic chemotherapy J Clin Oncol 2009 11 446 452 19064965 10.1200/JCO.2008.18.8151

[B12] Li P Wang SS Liu H Li N McNutt MA Li G Ding HG Elevated serum alpha fetoprotein levels promote pathological progression of hepatocellular carcinoma World J Gastroenterol 2011 11 4563 4571 10.3748/wjg.v17.i41.4563 22147961 PMC3226982

[B13] Zheng M Ruan Y Yang M Guan Y Wu Z The comparative study on ultrastructure and immunohistochemistry in AFP negative and positive hepatocellular carcinoma J Huazhong Univ Sci Technolog Med Sci 2004 11 547 559 10.1007/BF02911350 15791836

[B14] Fan WZ Yang JY Lü MD Transcatheter arterial chemoembolization plus percutaneous thermal ablation in large hepatocellular carcinoma: clinical observation of efficacy and predictors of prognostic factors [Article in Chinese] Zhonghua Yi Xue Za Zhi 2011 11 2190 2194 22094036

[B15] Luo JT Wei X Zhou HY Li Q Influencing factors for intrahepatic distant recurrence of liver cancer after radiofrequency ablation [Article in Chinese] Zhonghua Wai Ke Za Zhi 2009 11 1529 1531 20092738

[B16] Ikai I Arii S Kojiro M Ichida T Makuuchi M Matsuyama Y Nakanuma Y Okita K Omata M Takayasu K Yamaoka Y Reevaluation of prognostic factors for survival after liver resection in patients with hepatocellular carcinoma in a Japanese nationwide survey Cancer 2004 11 796 802 15305412 10.1002/cncr.20426

[B17] Reichman TW Bahramipour P Barone A Koneru B Fisher A Contractor D Wilson D Dela Torre A Cho KC Samanta A Harrison LE Hepatitis status, child-pugh classification, and serum AFP levels predict survival in patients treated with transarterial embolization for unresectable hepatocellular carcinoma J Gastrointest Surg 2005 11 638 645 10.1016/j.gassur.2004.11.002 15862257

[B18] Ha NB Ha NB Ahmed A Ayoub W Daugherty TJ Chang ET Lutchman GA Garcia G Cooper AD Keeffe EB Nguyen MH Risk factors for hepatocellular carcinoma in patients with chronic liver disease: a case–control study Cancer Causes Control 2012 11 455 462 10.1007/s10552-012-9895-z 22258434

[B19] Levi DM Tzakis AG Martin P Nishida S Island E Moon J Selvaggi G Tekin A Madrazo BL Narayanan G Garcia MT Feun LG Tryphonopoulos P Skartsis N Livingstone AS Liver transplantation for hepatocellular carcinoma in the model for end-stage liver disease era J Am Coll Surg 2010 11 727 734 735–736 10.1016/j.jamcollsurg.2010.01.007 20421039

[B20] Kobayashi M Hosaka T Ikeda K Seko Y Kawamura Y Sezaki H Akuta N Suzuki F Suzuki Y Saitoh S Arase Y Kumada H Highly sensitive AFP-L3% assay is useful for predicting recurrence of hepatocellular carcinoma after curative treatment pre- and postoperatively Hepatol Res 2011 11 1036 1045 10.1111/j.1872-034X.2011.00858.x 21883741

[B21] Jiang J Wu C Shen Y Xu B Zheng X Li X Xu N Clinical application of determining serum AFP-IgM complexes for diagnosis of small hepatocellular carcinoma Anticancer Res 2011 11 687 691 21378357

[B22] Xu WF Fei YM Zhou JK Shen HJ Chen XF Lv QQ Ding YY Significance of serum golgi protein 73 (GP73), alpha-fetoprotein (AFP) and lectin-reactive alpha-fetoprotein (AFP-L3) expresssion in primary hepatic carcinoma [Article in Chinese] Zhonghua Shi Yan He Lin Chuang Bing Du Xue Za Zhi 2011 11 286 288 22097609

[B23] Volk ML Hernandez JC Su GL Lok AS Marrero JA Risk factors for hepatocellular carcinoma may impair the performance of biomarkers: a comparison of AFP, DCP, and AFP-L3 Cancer Biomark 2007 11 79 87 17522429 10.3233/cbm-2007-3202

[B24] Yamamoto K Imamura H Matsuyama Y Kume Y Ikeda H Norman GL Shums Z Aoki T Hasegawa K Beck Y Sugawara Y Kokudo N AFP, AFP-L3, DCP, and GP73 as markers for monitoring treatment response and recurrence and as surrogate markers of clinicopathological variables of HCC Gastroenterol 2010 11 1272 1282 10.1007/s00535-010-0278-5 20625772

